# Cefazolin Improves Anesthesia and Surgery-Induced Cognitive Impairments by Modulating Blood-Brain Barrier Function, Gut Bacteria and Short Chain Fatty Acids

**DOI:** 10.3389/fnagi.2021.748637

**Published:** 2021-10-13

**Authors:** Ailin Luo, Shan Li, Xuan Wang, Zheng Xie, Shiyong Li, Dongyu Hua

**Affiliations:** Department of Anesthesiology, Tongji Hospital, Tongji Medical College, Huazhong University of Science and Technology, Wuhan, China

**Keywords:** postoperative cognitive dysfunction (POCD), cefazolin, blood-brain barrier, gut microbiota, short chain fatty acids

## Abstract

Emerging evidence suggests that anesthesia and surgery may induce gut dysbiosis. Gut dysbiosis leads to imbalance in circulating contents of microbiota-derived metabolites and disrupts the integrity of the blood-brain barrier (BBB), contributing to postoperative cognitive dysfunction (POCD). The composition of gut microbiota may be influenced by various antibiotics. However, how perioperative use of antibiotics affects POCD needs more explorations. In the present study, we explored the effect of cefazolin, a common antibiotic used in perioperative period, on cognitive function, BBB integrity, gut bacteria and short chain fatty acids (SCFAs), a group of widely studied metabolites in aged mice, using 18-month-old male mice. Significant BBB disruptions and decreased levels of tight junction proteins, zonula occludens-1 (ZO-1) and Occludin (OCLN) were seen in the mice of POCD model. Cefazolin treatment attenuated these changes induced by anesthesia and surgery. Furthermore, cefazolin reversed the changes in several fecal bacteria (β*-*, γ/δ*-*, ε*-Proteobacteria*, and Bacteroidetes) as determined by qPCR tests. Analysis of plasma SCFAs showed that almost all types of SCFAs were reduced in POCD and cefazolin administration reversed the changes in expression of the two most abundant SCFAs (acetic and propionic acids). In conclusion, this study demonstrated that cefazolin improved POCD. Mechanistically, cefazolin suppressed the disruption of BBB, gut microbiota or SCFAs, thereby ameliorating POCD.

## Introduction

Postoperative cognitive dysfunction (POCD), a serious complication after anesthesia and surgery in the elderly, causes delayed recovery, increased mortality and social burden (Hovens et al., 2012). However, the exact mechanism of POCD remains unclear. Recent studies have indicated that anesthesia and surgery may cause an imbalance of gut microbiota, termed gut dysbiosis, which plays a critical role in POCD (Luo et al., 2019; Lin et al., 2020; Czok et al., 2021). Moreover, prebiotics or probiotics could alter gut microbiota and its metabolites, thereby improving cognitive impairments (Yang et al., 2018; Mao et al., 2021). Therefore, gut microbiota and associated metabolites may be promising preventive and therapeutic strategies for POCD.

The composition of gut microbiota has been mainly associated with age, lifestyles (exercises or eating habits), environmental toxins and drugs. Antibiotic cocktails for depleting microbiota have been used to explore the therapeutic effect of prebiotics, probiotics or fecal microbiota transplantation on POCD (Jiang et al., 2019; Wen et al., 2020; Mao et al., 2021). The use of perioperative antibiotics is a routine practice in clinical surgery. However, the effect of perioperative use of antibiotics on gut microbiota and cognitive function is less understood. Recently, Liang et al. firstly elucidated that cefazolin administration based on clinical practice (30 min before surgery and 5 days after surgery) could attenuate surgery-induced cognitive impairments and neuroinflammation in young mice. Moreover, cefazolin treatment without surgery may cause dysbiosis and cognitive deficits (Liang et al., 2018). In their study, POCD model was constructed in young mice (6- to 8-week-old). It is well-known that age is an independent factor of POCD (Luo et al., 2019). Therefore, the influence of cefazolin on cognitive function, gut microbiota and associated metabolites in aged mice is worthy of further exploration.

Mounting evidence suggested that both neuroinflammation and disruption of the brain-blood barrier (BBB) play critical roles in the pathogenesis of POCD (Luo et al., 2019). BBB prevents neurotoxins entry into the brain and increased hippocampal BBB leakage may exacerbate neuroinflammation and cognitive impairment (Fulop et al., 2019). Numerous preclinical studies showed that anesthesia and surgery may disrupt hippocampal BBB function (Li et al., 2016; Cao et al., 2018, 2019). Unfortunately, advanced age is also associated with BBB leakage. Therefore, enhancement of BBB function could be beneficial to POCD. Several studies demonstrated that antibiotic-induced alterations of gut microbiota influenced BBB permeability (Wu Q. et al., 2020; Sun et al., 2021). Wen et al. (2020) also reported in a splenectomy–induced POCD model that diminishment of gut microbiota by antibiotics mix would decrease the expression of tight junction protein, resulting in disruption of BBB. Different with antibiotics mix, cefazolin is an antibiotic for preventing infections and it attenuates surgery-induced neuroinflammation, whether cefazolin affects the BBB function through gut microbiota should be elucidated.

Herein, we investigated the effect of cefazolin on anesthesia and surgery-induced cognitive impairments and BBB function in aged mice. In addition, we explored cefazolin-induced alterations of gut bacteria and important metabolites, short-chain fatty acids (SCFAs), and analyze their possible effect on BBB function and cognitive performance in the POCD model.

## Materials and Methods

### Animals

This study was approved by the Experimental Animal Care and Use Committee of Tongji Hospital, Tongji Medical College, Huazhong University of Science and Technology. A total of 84 male C57BL/6J mice (18-month-old, 32–36 g) were used in this study. All mice were purchased from the Laboratory Animal Centre of Tongji Medical College, Huazhong University of Science and Technology. Mice were housed in a controlled room (12 h light/dark cycle, temperature: 22 ± 2°C, humidity: 60 ± 5%) with water and food *ad libitum*.

### Study Design

#### Experiment I

**FIGURE 1:**
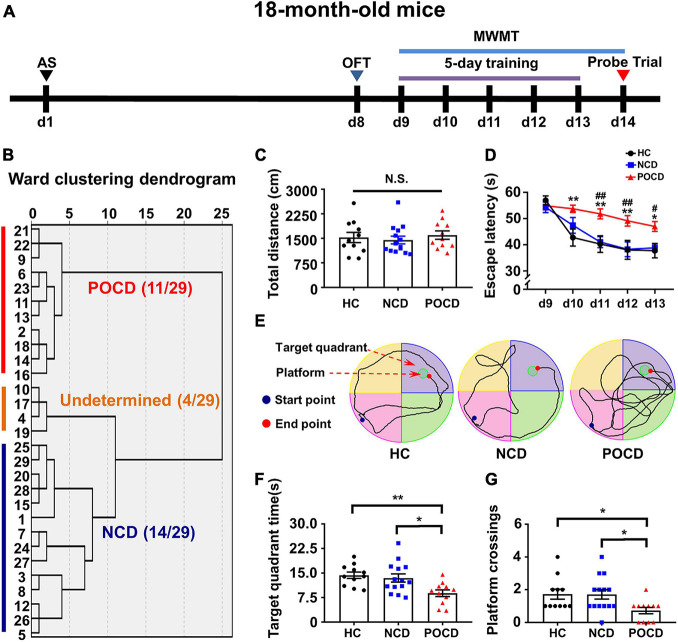
Anesthesia and surgery induced post-operative cognitive impairment in aged mice. **(A)** The schematic of the Experiment I. Anesthesia and surgery (AS) were performed on day 1 (d1). OFT was performed on day 8 followed by recovery period of 1 week. On day 9–14, mice were trained for MWMT, training test was performed on day 9–13 and spatial probe test was performed on day 14. Then, clustering analysis was performed and tissue samples were collected. **(B)** Dendrogram of hierarchical clustering analysis. A total of 29 mice after AS were divided into undetermined, NCD and POCD phenotypes by clustering analysis of MWMT results. **(C)** Total distance in OFT. **(D)** Escape latency: POCD vs. HC, *P < 0.05 or **P < 0.01; POCD vs. NCD, ^#^*P* < 0.05 or ^##^*P* < 0.01. **(E)** Representative trace graphs of HC, NCD, and POCD mice in MWMT on day 13 (the last day of training). **(F)** Target quadrant time: *P < 0.05 or **P < 0.01. **(G)** Platform crossing: POCD vs. HC, *U* (Mann-Whitney value) = 25.50, *P < 0.05; POCD vs. NCD, *U* = 35, *P < 0.05. Data are shown as mean ± S.E.M. (HC: *n* = 11, NCD: *n* = 14, POCD: *n* = 11). AS, anesthesia and surgery; d, day; HC, healthy control; MWMT, Morris water maze test; NCD, non-POCD; N.S., not significant; OFT, open field test; POCD, post-operative cognitive dysfunction.

**FIGURE 2:**
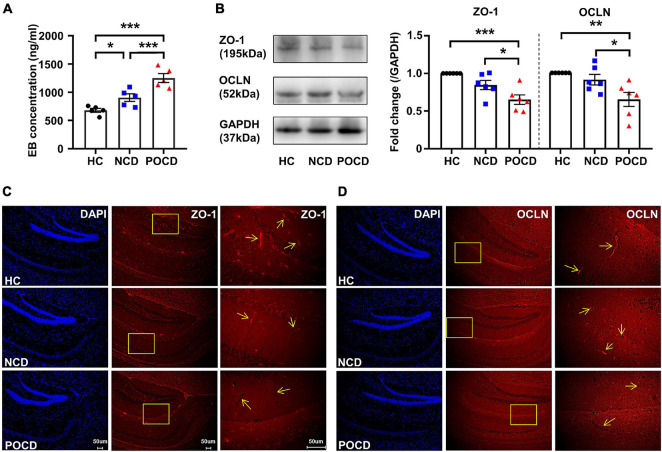
Increased blood-brain barrier (BBB) permeability and decreased expressions of tight junction proteins in POCD. **(A)** Hippocampal Evans blue (EB) concentrations of mice in HC, NCD, and POCD groups. **(B)** Representative bands and quantitation of ZO-1 and OCLN proteins in the hippocampi (relative to GAPDH). **(C)** Representative images of DAPI and ZO-1 in the hippocampus for immunofluorescence. **(D)** Representative images of DAPI and OCLN in the hippocampus for immunofluorescence. Data are shown as mean ± S.E.M. (for EB assay, *n* = 5 in each group; for western blot and immunofluorescence, *n* = 6 in each group). *P < 0.05, **P < 0.01, or ***P < 0.001. EB, Evans blue; GAPDH, glyceraldehyde phosphate dehydrogenase; HC, healthy control; NCD, non-POCD; N.S., not significant; OCLN, occludin; POCD, post-operative cognitive dysfunction; ZO-1, zonula occludens 1.

Forty mice were randomly divided into two groups: healthy control (HC) group (*n* = 11) and anesthesia and surgery (AS) group (*n* = 29). The intramedullary fixation for open tibial fracture with inhalation anesthesia was performed was performed in the AS group on day 1 as previously reported (Sun et al., 2017). No intervention was made in the HC group on day 1. After 6 days of recovery, behavioral tests were performed on days 8–14 (Figure 1A). A cluster analysis based on the Ward method was used to categorize the AS group into POCD, non-cognitive dysfunction (NCD) and the undetermined phenotype (Figure 1B). Comparisons among HC, NCD, and POCD groups were made as shown in Figures 1, 2.

#### Experiment II

Cefazolin sodium (HY-B1078, MCE, NJ, United States) was dissolved into in a vehicle, 0.9% saline. To determine the effect of cefazolin on POCD in aged mice, 44 mice were randomly divided into 4 groups: HC + vehicle group, HC + cefazolin group, AS + vehicle group, and AS + cefazolin group (*n* = 11 in each group). Cefazolin at 400 mg/kg was injected intraperitoneally 0.5 h before the surgery and once each day for 4 days post-surgery, following a previous study (Liang et al., 2018). Then, behavioral tests were performed on days 8–14.

In the two experiments, each group contained 11 mice. Five mice were randomly selected to check BBB permeability of using an Evans blue (EB) dye assay. The remaining six mice were sacrificed and their brains were collected immediately. One half of the brains was stored at −80°C for Western blot analysis and the other half was fixed in 4% paraformaldehyde for immunohistochemistry.

### Open Field Test

Open field test (OFT) is frequently used to evaluate anxiety-related behavior and locomotor activity. Considering that the fracture surgery may cause different conditions of bone injury and recovery, we used the OFT to detect the difference in locomotor activity between groups with and without surgery as previously reported (Zhan et al., 2019). OFT was performed on day 8. Briefly, mice were placed in a white box (40 cm × 40 cm × 40 cm) and allowed to move freely for 5 min. The box was cleaned with 75% ethanol between the tests. The total distance (cm) covered by mice was recorded and analyzed by a video tracking system (Zongshi Technology, Beijing, China).

### Morris Water Maze Test

Morris water maze test (MWMT) was conducted on days 9–14 to assess spatial learning and reference memory. MWMT consisted of training trails (for 5 days) and probe tests (for 1 day). A white circular pool (diameter 120 cm, height 50 cm) with water (temperature: 23 ± 1°C) was divided into four quadrants. A hidden white platform (10 cm in diameter) was located in the target quadrant and 1 cm below the water surface. Each mouse was placed at a fixed position in every quadrant and given 1 min to locate the platform. Mice were guided to the platform if they failed to find the platform. Mice were allowed to stay on the platform for 15 s. This procedure lasted for 5 days and escape latency (time taken to locate the platform) was recorded using a video tracking system (Zongshi Technology, Beijing, China). Then, the platform was removed to perform a probe test on day 14. Mice were placed into the pool from each quadrant for 1 min. The number of platform crossing times and the time spent in the target quadrant were recorded to assess the reference memory.

### Evans Blue Dye Extravasation Assay

Blood-brain barrier permeability was evaluated by measuring EB dye extravasation in mice hippocampi (*n* = 5 for each group) as previously reported (Hu et al., 2014). Briefly, 2% EB dye (abs47002009, Absin, Shanghai, China) was dissolved in saline and injected slowly through the tail vein (4 ml/kg). The EB dye was allowed to circulate for 3 h. Then, the animals were perfused with ice-cold phosphate-buffered saline (PBS) and the hippocampi were separated. Each hippocampus was immersed in 1 ml of formamide for 72 h at room temperature. The samples were centrifuged at 10,000 *g* for 10 min. The absorbance of each sample was measured at 620 nm and the concentration of the EB dye was determined by the standard curve of the EB dye in formamide with different concentration gradients (0, 25, 125, 250, 500, 750, 1,000, 1,500, and 2,000 ng/ml).

### Western Blot

**FIGURE 3:**
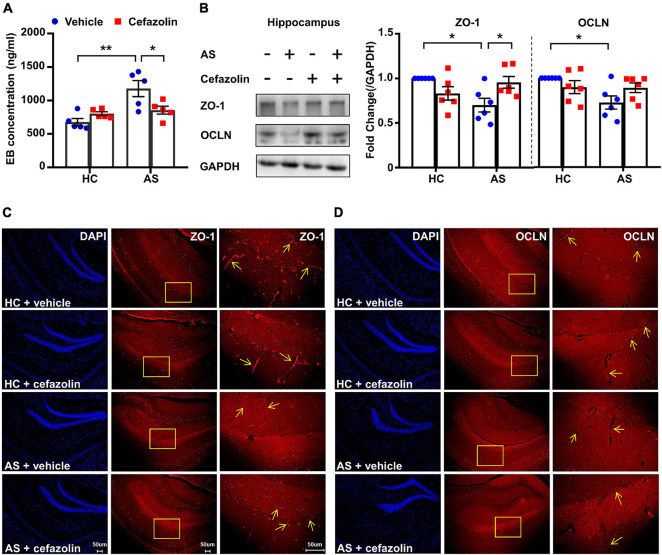
Perioperative use of cefazolin reversed the alterations of BBB permeability and tight junction proteins in the AS group. **(A)** Hippocampal EB concentration of mice in the four groups. **(B)** Representative bands and quantitation of ZO-1 and OCLN proteins in the hippocampi (relative to GAPDH). **(C)** Representative images of DAPI and ZO-1 in the hippocampus for immunofluorescence. **(D)** Representative images of DAPI and OCLN in the hippocampus for immunofluorescence. Data are shown as mean ± S.E.M. (for EB test, *n* = 5 in each group; for western blot and immunofluorescence, *n* = 6 in each group). *P < 0.05 or **P < 0.01. AS, anesthesia and surgery; EB, Evans blue; GAPDH, glyceraldehyde phosphate dehydrogenase; HC, healthy control; NCD, non-POCD; N.S., not significant; OCLN, occludin; POCD, post-operative cognitive dysfunction; ZO-1, zonula occludens 1.

The hippocampi were homogenized in ice-cold radioimmunoprecipitation assay (RIPA) lysis buffer (Boster, Wuhan, China) containing protease and phosphatase inhibitor. The homogenized tissues were centrifuged at 10,000 *g* for 15 min at 4°C. The protein concentrations were measured using a bicinchoninic acid (BCA) protein assay kit (Boster, Wuhan, China). The samples were loaded and run on 10% sodium dodecyl sulfate polyacrylamide gel electrophoresis (SDS-PAGE). The separated proteins were transferred to poly-vinylidene fluoride (PVDF) membranes. The bands were blocked with 5% bovine serum albumin (BSA) for 1 h at room temperature and incubated with anti-Zonula occludens-1 (ZO-1, 1:1,000, AF5145, Affinity Biosciences, Cincinnati, OH, United States), anti-Occludin (OCLN, 1:1,000, DF7504, Affinity Biosciences, Cincinnati, OH, United States) overnight at 4°C. After warming and washing with Tris-buffered saline with Tween-20 (TBST), the bands were then incubated with horseradish peroxidase-conjugated secondary antibody goat anti-rabbit IgG (1:5,000, BA1055, Boster, Wuhan, China) for 2 h at room temperature. The bands were washed four times 8 min with TBST before imaging. For visualization, the bands were developed using enhanced chemiluminescence (ECL, Aspen, Wuhan, China) and detected under a ChemiDoc XRS chemiluminescence imaging system (Bio-Rad, Hercules, CA, United States). The levels of each protein were standardized to 1 in the HC group (Figure 2) and the HC + vehicle group (Figure 3). Original bands were shown in the Supplementary Material.

### Immunofluorescence

To assess the alterations of tight junction proteins in the hippocampus, IF staining was performed following a previous report (Li et al., 2021). The brain tissues (half, *n* = 6) were embedded in paraffin and sliced into 4 μm-thick sections. Briefly, after dewaxation and rehydration, the sections were immersed in 0.01 mol/l sodium citrate buffer (pH 6.0) and heated by a microwave oven to induce antigen retrieval. After washing, the sections were incubated with normal goat serum at 4°C overnight. Then, primary antibodies anti-ZO-1 (1:200) and anti-OCLN (1:100) were added and incubated at 4°C for 24 h. The sections were washed and incubated with goat anti-rabbit polyclonal secondary antibody (1:100, A23620, Abbkine Scientific Co., Ltd, Wuhan, China) for 2 h at room temperature. Then, 4,6-diamidino-2-phenylindole (DAPI, BMD00063, Abbkine Scientific Co., Ltd, Wuhan, China) was added for 10 min at room temperature. The operations were performed in a dark room. The sections were observed using a fluorescence microscope (DM2500, Leica, Germany).

### DNA Extraction of Fecal Samples

DNA was extracted from the fresh stools of mice by using a QIAamp Fast DNA stool Mini Kit (Qiagen Inc., Valencia, CA, United States). Briefly, fecal samples (180–220 mg) were transferred to 2-mL tubes and placed on ice. Then, the samples were lysed in 1 ml InhibitEX buffer and vortex-mixed continuously for 1 min. The samples were heated at 95°C for 5 min and centrifuged at 20,000 *g* for 1 min to pellet stool particles. After centrifugations, 600 μL of the supernatant from each lysate was transferred into a 2-mL tube containing 25 μL proteinase K followed by adding 600 μl Buffer AL. The lysates were incubated at 70°C for 10 min and 600 μL of absolute ethanol was added. All the lysates were applied to the QIAamp spin columns and centrifuged at 20,000 *g* for 1 min. Then, the columns were washed (20,000 *g*, 3 min) twice by adding 500 μl wash buffer (Buffer AW1 and AW2). After washing, 200 μl Buffer ATE was added directly onto the QIAamp membrane of each column to elute DNA (20,000 *g*, 1 min). DNA concentrations and quality (A260/A280 and A260/A230) were measured using a NanoDrop ND-1000 spectrophotometer (NanoDrop Technologies Inc., DE, United States). DNA samples was stored at −20°C.

### Quantitative Polymerase Chain Reaction

**FIGURE 4:**
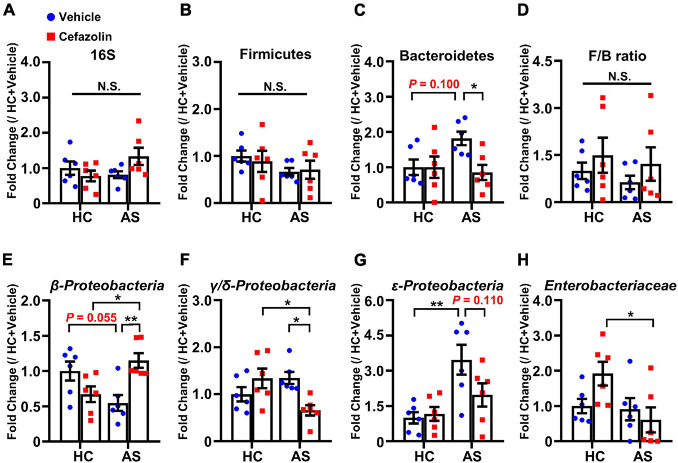
Cefazolin changed the composition of fecal bacteria in the HC and AS groups. **(A)** 16S rRNA, **(B)** Firmicutes, **(C)** Bacteroidetes, **(D)** F/B ratio, **(E)** β*-Proteobacteria*, **(F)** γ/δ*-Proteobacteria*, **(G)** ε*-Proteobacteria*, and **(H)** *Enterobacteriaceae*. Data are shown as mean ± S.E.M. (*n* = 6 in each group). *P < 0.05 or **P < 0.01. AS: anesthesia and surgery; HC: healthy control; N.S.: not significant.

Bacteroidetes and Firmicutes are the most abundant bacterial phyla in the gut, and a higher Firmicutes-/Bacteroidetes (F/B) ratio is usually implicated in dysbiosis in various diseases (Magne et al., 2020). Furthermore, Proteobacteria or *Enterobacteriaceae* are considered important signatures of dysbiosis (Shin et al., 2015; Rivera-Chavez et al., 2017). Thus, relative contents of 16S (total bacteria), Firmicutes, Bacteroidetes, β*-*, γ/δ*-*, ε*-Proteobacteria*, and *Enterobacteriaceae* were detected using qPCR as a previous study of Jiang et al. (2018). Briefly, qPCR for several gut bacteria (Figures 4A–H) was carried out with fecal total DNA (0.1 μg). The thermal cycling conditions were 95°C for 30 s, followed by 45 cycles at 95°C for 5 s (denaturation) and 63°C for 30 s (annealing and extension). The bacterial population level relative to 16S ribosomal RNA was calculated. The relative abundance of each bacterium was calculated as the ratio of the population level of each bacterium in each feces to the population level of each bacterium in the highest one of all the feces (Jang et al., 2018). To facilitate the comparisons, we further standardized the mean of relative abundance of each bacterium in the HC + vehicle group to 1. The results were finally presented as fold changes relative to the HC + vehicle group. Primers of each bacterium were obtained from previous studies (Jang et al., 2018; Chen et al., 2020).

### Detection of Short Chain Fatty Acids

The concentrations of total SCFAs, acetic acid, propionic acid, isobutyric acid, butyric acid, isovaleric acid, valeric acid and hexanoic acid in plasma were measured for SCFA analysis. The plasma samples of mice (*n* = 6 for each group) were collected. Plasma samples were thawed on ice and 150 μl of plasma was transferred into 2 ml micro-tubes for further analysis. About 900 μl of phosphoric acid was added to each sample. After centrifugation at 14,000 *g* for 10 min, 800 μl of the supernatant was obtained and an equal amount of ethyl acetate was added. After mixing for 2 min, the samples were centrifuged at 14,000 *g* for 10 min. About 600 μl of the upper organic phase was taken and 500 μM (final concentration) of 4-methylvaleric acid was added as an internal standard. SCFA analysis was performed using 7890A/5975C gas chromatography-mass spectrometry (GC-MS, Agilent, United States). The supernatant was separated using a Agilent DB-WAX capillary column (30 m × 0.25 mm × 0.25 μm). The injection volume was 1 μL per sample and split mode was set at a ratio of 10:1. The temperature program was as follows: the initial oven temperature was held at 90°C, elevated to 120°C at a rate of 10°C/min, increased to 150°C at a rate of 5°C/min, and finally increased to 250°C at a rate of 20°C/min, and held at 250°C for 2 min. The flow rate of the carrier gas (Helium) was set at 1 mL/min through the column. In these sample queues, a quality control (QC) sample was inserted between a certain number of experimental samples to evaluate the stability and repeatability of the system. The temperatures of the inlet, transfer interface, electron impact ion source and the quadrupole were 250, 250, 230, and 150°C, respectively. The mass range in the full-scan or selected ion monitor (SIM)-scan mode was applied for electron impact ionization (70 eV). The peak area was used to calculate SCFA concentrations. Raw GC-MS data were processed using MSD ChemStation software (Agilent Technologies, United States) for peak area and retention time integration. Quantitative analysis was based on an established standard curve of each SCFA.

### Statistical Analysis

**FIGURE 5:**
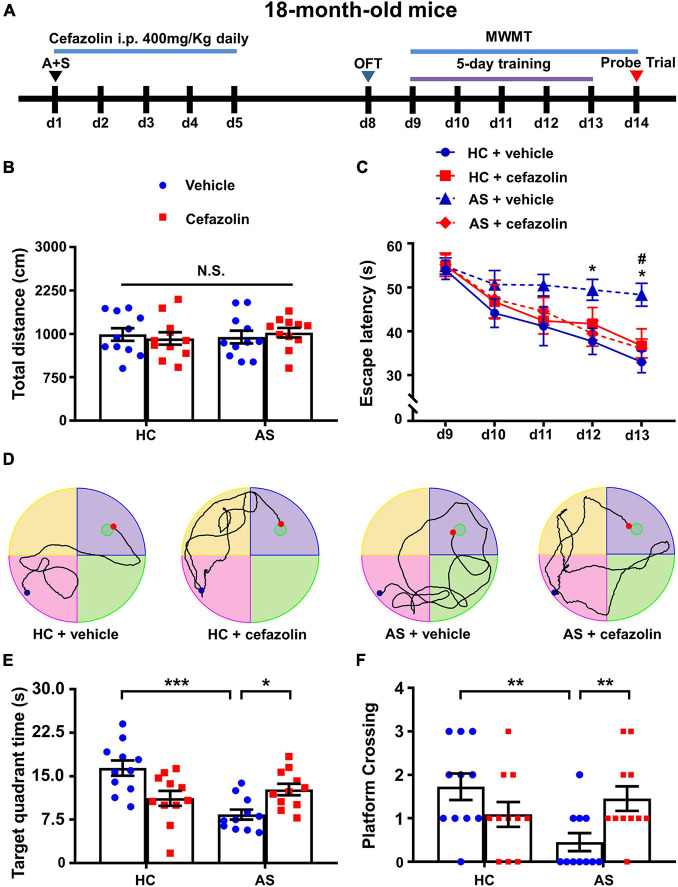
Perioperative use of cefazolin attenuated cognitive dysfunction induced by AS. **(A)** The schematic of Experiment II. AS was performed on day 1, and cefazolin (400 mg/kg, i.p.) was administered at 0.5 h before the surgery (d1) and once each day for 4 days (d2–d5). OFT was performed on day 8. MWMT was performed on day 9–14. **(B)** Total distance in OFT. **(C)** Escape latency of training: AS + Vehicle vs. HC + Vehicle, *P < 0.05; AS + Vehicle vs. AS + Cefazolin, ^#^*P* < 0.05. **(D)** Representative traces graphs (the last day of training) of mice in the four groups. **(E)** Target quadrant time: *P < 0.05 or ***P < 0.001. **(F)** Platform crossing: AS + Vehicle vs. HC + Vehicle, *U* = 19, **P < 0.05; AS + Vehicle vs. AS + Cefazolin, *U* = 23.50, **P < 0.05. Data are shown as mean ± S.E.M. (*n* = 11 in each group). AS, anesthesia and surgery; d, day; HC, healthy control; MWMT, Morris water maze test; N.S., not significant; OFT, open field test.

The means of escape latency at the last day of training, times of platform crossing and durations in the target quadrant were used for hierarchical cluster analysis. Cluster analysis was performed using the Ward method based on the square Euclidean distance by SPSS 22.0. Results from Western blotting and behavioral tests are shown as the mean ± standard error of the mean (SEM). Distribution normality was assessed by the Kolmogorov-Smirnov test and homogeneity of variance was assessed by the Levene test. Platform crossing (Figures 1G, 5F) was analyzed by non-parametric tests (Kruskal-Wallis test or Scheirer-Ray-Hare test) followed by Mann-Whitney *post hoc* tests. Escape latency (Figures 1D, 5C) was analyzed by two-way repeated-measures ANOVA. The other comparisons among groups were analyzed by one-way ANOVA or two-way ANOVA followed by Bonferroni correction. If the interaction factor was significant in two-way ANOVA, a simple effect was performed instead of *post hoc* test. A *P*-value < 0.05 was considered statistically significant. Data were analyzed by GraphPad Prism software version 8.0 or R companion package (for Scheirer-Ray-Hare test) of R version 4.1.0.

## Results

### Anesthesia and Surgery Induced Postoperative Cognitive Dysfunction in 18-Month-Old Male Mice

The schematic of Experiment I is shown in Figure 1A. A total of 29 mice were grouped into POCD, non-POCD (NCD) and undetermined group based on hierarchical cluster analysis (Figure 1B). The result showed that 11 mice were assigned to the POCD group and 14 mice were assigned to the NCD group. There was no significant change in the total distance covered by mice in OFT in the HC, NCD and POCD groups (*F*_2_,_33_ = 0.332, *P* = 0.720), suggesting similar mobility among the three groups (Figure 1C). MWMT results showed that the escape latency was significantly changed with time (*F*_4_,_132_ = 19.88, *P* < 0.001) and among groups (*F*_2_,_33_ = 10.45, *P* < 0.001). Escape latency was higher in the POCD group compared to the HC group (on day 10, 11, 12, and 13) and NCD group (on day 11, 12, and 13) (Figure 1D). Representative MWMT trace graphs for day 13 showed differential behavioral performance among the three groups (Figure 1E). In the probe test, target quadrant time (*F*_2_,_33_ = 6.358, *P* = 0.005) and platform crossings (*H* = 8.119, *df* = 2, *P* = 0.017) were significantly lower in the POCD group than in the HC and NCD groups (Figures 1E,F). MWMT results suggested that AS (tibial fracture) induced POCD in nearly 40% of aged mice.

### Disruption of the Blood-Brain Barrier and Decrease in Tight Junction Proteins in the Postoperative Cognitive Dysfunction Model

Previous studies demonstrated a disruption of the BBB in the POCD model with tibial fracture (Zhang et al., 2016; Wu T. et al., 2020). In the present study, we also evaluated changes in BBB permeability. The mean concentration of the EB dye in the hippocampi was significantly different among the three groups (*F*_2_,_12_ = 21.33, *P* < 0.001). EB concentration of the POCD group was significantly higher than that in the HC and NCD groups (Figure 2A). BBB disruption has been associated with the loss of tight junctions. Western blotting and IF was used to detect the expression of ZO-1 and OCLN, markers of tight junctions. The levels of ZO-1 (*F*_2_,_15_ = 11.99, *P* < 0.001) and OCLN (*F*_2_,_18_ = 7.470, *P* = 0.006) were decreased in the POCD group compared with the NCD and HC groups (Figure 2B), suggesting a loss of tight junctions in the POCD model. IF staining in hippocampi showed that ZO-1 and OCLN were mainly located in brain microvessels and the results were comparable among the groups (Figures 2C,D).

### Perioperative Use of Cefazolin Attenuated Anesthesia and Surgery-Induced Cognitive Deficits

To determine whether antibiotics affected the cognitive performance in the POCD model, cefazolin (400 mg/kg, i.p.) was given at 0.5 h before the surgery and 4 days after surgery (Figure 5A). There was no significant change in the total distance covered by mice in OFT among the HC + vehicle, HC + cefazolin, AS + vehicle, and AS + cefazolin groups (Figure 5B). Statistical effect analysis of escape latency suggested that major effects of time, interaction and subject factors were significant but major effect of group was not significant (Group: *F*_3_,_40_ = 3.895, *P* = 0.016; Time: *F*_4_,_160_ = 20.33, *P* < 0.001; Interaction: *F*_12_,_160_ = 0.918, *P* = 0.530; Subject: *F*_40_,_160_ = 2.440, *P* < 0.001). Also, the interaction factors were significant in analysis of the target quadrant time (Interaction: *F*_1_,_40_ = 17.89, *P* < 0.001) and platform crossings (Interaction: *H* = 8.191, *df* = 1, *P* = 0.007). Thus, simple effect analyses were performed. Cefazolin administration in the AS group significantly decreased escape latency on day 13 (*P* < 0.05) and increased the target quadrant time (*P* < 0.05) and platform crossing (*P* < 0.01) compared with AS + vehicle group (Figures 5C,E,F). However, cefazolin administration in the HC group slightly increased the escape latency and decreased the target quadrant time and platform crossings compared with HC + vehicle group, Figures 5C,E,F). These results indicated that cefazolin could improve POCD, but induce or aggravate cognitive declines in healthy aged mice. Representative MWMT trace graphs for the four groups are shown in Figure 1D.

### Perioperative Use of Cefazolin Improved Blood-Brain Barrier Disruption and Increased the Expression of Tight Junction Proteins

Interactions between surgery and cefazolin treatment were also significant in the analysis of EB concentrations (Interaction: *F*_1_,_16_ = 9.322, *P* = 0.008), protein levels of ZO-1 (Interaction: *F*_1_,_20_ = 11.32, *P* = 0.003) and OCLN (Interaction: *F*_1_,_20_ = 5.165, *P* = 0.034). Simple effect analysis showed that perioperative use of cefazolin in the AS group decreased the hippocampal concentration of the EB dye compared to the AS + vehicle (*P* < 0.05; Figure 3A), suggesting that cefazolin could reverse BBB disruptions in POCD. Western blot and IF results revealed that cefazolin administration in the AS group increased ZO-1 and OCLN (not statistical significant) levels in hippocampal microvessels (Figures 2B–D), suggesting that cefazolin might attenuate the loss of tight junction induced by AS. However, when compared with the HC + vehicle and HC + cefazolin groups, cefazolin alone (HC + cefazolin group) did not induce significant effects on BBB permeability and the expression of tight junction proteins.

### Perioperative Use of Cefazolin Reversed Anesthesia and Surgery-Induced Alterations in Gut Bacteria

The effects of cefazolin on the changes in gut microbiota were explored. qPCR was performed to detect the relative contents of gut bacteria. Also, interaction effects were almost present in all analyses of bacteria. AS and cefazolin (both in the HC and AS groups) decreased the relative content of 16S, but no statistical significance was found (Figure 4A). AS decreased the content of β*-Proteobacteria* (*P* = 0.055) and increased the content of Bacteroidetes (*P* = 0.100), γ/δ*-Proteobacteria* (*P* = 0.179), and ε*-Proteobacteria* (*P* < 0.01) compared with HC + vehicle group (Figures 4C,E–G). However, perioperative use of cefazolin reversed the alterations in these bacteria, suggesting that cefazolin modulated gut microbiota. Because cefazolin was injected intraperitoneally (not by oral gavage), the effect of cefazolin on microbiota might be associated with inhibiting gut inflammation, which modulated the gut microenvironment. Besides, perioperative use of cefazolin increased the F/B ratio with no statistical significance (Figure 4D). Cefazolin alone decreased β*-Proteobacteria* and increased γ/δ*-Proteobacteria* (not significant) and *Enterobacteriaceae* content (Figures 4E,F,H). This might be because some strains of *Enterobacteriaceae* produce extended-spectrum β-lactamases, which cause their abnormal growth after cefazolin treatment.

### Perioperative Use of Cefazolin Reversed Anesthesia and Surgery-Induced Alterations in Short Chain Fatty Acids

**FIGURE 6:**
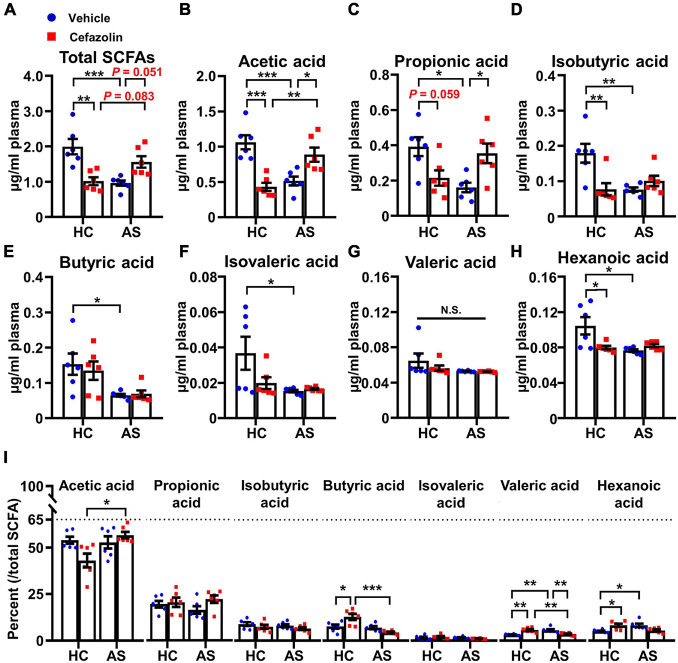
Cefazolin changed the contents of plasma SCFAs in the HC and AS groups. **(A)** Total SCFAs, **(B)** Acetic acid, **(C)** Propionic acid, **(D)** Isobutyric acid, **(E)** Butyric acid, **(F)** Isovaleric acid, **(G)** Valeric acid, **(H)** Hexanoic acid, and **(I)** The percent of each SCFA relative to total SCFAs. Data are shown as mean ± S.E.M. (*n* = 6 in each group). *P < 0.05, **P < 0.01 or ***P < 0.001. AS, anesthesia and surgery; HC, healthy control; N.S., not significant; SCFAs, short chain fatty acids.

To determine the effects of cefazolin on the plasma contents of SCFAs, GC-MS was performed. Interaction effects existed in the SCFAs except butyric acid and valeric acid. The further analyses showed that AS significantly decreased total SCFAs and almost all types of SCFAs compared with the AS + vehicle and HC + vehicle groups (Figures 6A–H). Perioperative use of cefazolin increased the content of total SCFAs, acetic acid and propionic acid (Figures 6A–C), suggesting that acetic and propionic acids might be associated with the beneficial effect of cefazolin on cognitive performance. Notably, cefazolin alone significantly decreased the content of total SCFAs and most of SCFAs, except butyric acid and valeric acid (Figures 6A–H), indicating that cefazolin might influence SCFAs-producing bacteria in healthy aged mice. Analysis of the constituent ratio of each SCFA showed that acetic, propionic, butyric, and isobutyric acids were more abundant than the other SCFAs (Figure 6I). Perioperative use of cefazolin did not change the constituent ratio of the four SCFAs. However, cefazolin alone increased the ratio of butyric acid and decreased the ratio of acetic acid.

## Discussion

In the present study, we constructed a POCD model in 18-month-old mice through performing isoflurane anesthesia and tibial fracture. The results obtained from behavioral tests, quantitative analysis of EB dye concentration, Western blotting and IF verified BBB disruptions in the POCD model of aged mice. However, perioperative administration of cefazolin (0.5 h before surgery and 4 days post-surgery) significantly decreased BBB permeability and improved cognitive deficit induced by anesthesia and surgery. Furthermore, cefazolin reversed the abnormal changes in the relative abundance of several gut bacteria (Bacteroidetes, β*-Proteobacteria*, γ/δ*-Proteobacteria*, and ε*-Proteobacteria*) and the concentrations of plasma SCFAs. A previous study has demonstrated that the probiotic Lactobacillus and the component of SCFAs (sodium butyrate) could prevent POCD in aged mice via modulation of the BBB function (Wen et al., 2020). Collectively, these results indicated that cefazolin attenuated anesthesia and surgery-induced BBB damage by altering the gut bacteria abundance and associated metabolites, consequently decreasing the occurrence of POCD in aged mice.

A previous clinical study reported POCD in 41.4% of elderly patients at hospital discharge after non-cardiac surgery (Monk et al., 2008). To investigate the incidence of POCD in the aged mice model, we performed a cluster analysis based on behavioral results and classified the mice undergoing surgery into NCD, POCD and undetermined phenotypes. The results showed that 37.9% (11/29) of aged mice developed a POCD phenotype (Figure 1B). This incidence was consistent with the rate mentioned above. Notably, our incidence (37.9%) was a little lower than the actual incidence because of the undetermined (4/29) group obtained after cluster analysis. Further categorization of this group into POCD or NCD phenotypes proved difficult because the MWMT results of this group were intermediate.

Blood-brain barrier, an important gateway of the central nervous system, restricts the transport of molecules (e.g., cytokines) into the brain. Several studies in rodents demonstrated that inhalation anesthesia (isoflurane and sevoflurane) and/or surgery induced the BBB disruption (He et al., 2012; Li et al., 2016; Wen et al., 2020). Tight junctions, including ZO-1, OCLN, and claudin-5, are important components of the BBB. It was evidenced that alteration of tight junctions is implicated in the development of POCD (Hu et al., 2014; Cao et al., 2018; Wen et al., 2020). Our study also confirmed the alteration of BBB and tight junctions in the POCD model of aged mice. The results showed disruptions of BBB and a decrease in tight junction proteins (ZO-1 and OCLN) in the hippocampus of the POCD phenotype. However, significant difference was also found between the healthy control group and NCD phenotype. These indicated that BBB disruption might be a prerequisite for POCD.

Clinical administration of antibiotics to infections in the perioperative period is common. Cefazolin, a first-generation cephalosporin, is widely used as an effective and sensitive antibiotic prophylaxis in orthopedic surgery because of its rapid bone concentrations (Backes et al., 2017). However, its effect on POCD is rarely reported. Liang et al. (2018) firstly demonstrated that administration of cefazolin (*in vitro* and *in vivo*) may exert a direct anti-inflammatory effect and ameliorate cognitive impairment induced by isoflurane anesthesia and exploratory laparotomy. We further investigated the effect of cefazolin on BBB function and tight junctions in aged mice in the present study. The results showed that cefazolin significantly improved cognitive impairment and reversed the alterations in BBB function and tight junction proteins in the POCD model. Behavioral performance tended to be worse in the cefazolin without surgery group but there was no statistical significance. However, this tendency was not observed in the BBB function and tight junctions. The inconsistency in behavioral and molecular biological results may be explained by two possible reasons. Firstly, we used aged mice (18-month-old) to construct the POCD model and age-related BBB disruption might obscure the effect of cefazolin alone on BBB function. Secondly, BBB disruption in the aged brain allowed the entry of peripheral cytokines in the brain and caused neuroinflammation. Cefazolin alone might not change the BBB function but induce cognitive impairment through the other peripheral mechanisms, such as promoting the translocation of bacteria or endotoxin, resulting in peripheral inflammation.

Accumulating evidence suggested that abnormal gut microbiota may play a critical role in age-related cognitive impairment, including POCD (Ticinesi et al., 2019). Several reports, including one of our previous studies, have assessed the alterations of gut microbiota composition via 16S rRNA gene sequencing (Yang et al., 2018; Jiang et al., 2019; Zhan et al., 2019). It was found that dozens of commensal bacteria were changed in POCD. These bacteria mainly belonged to four phyla, including Firmicutes (*Erysipelotrichaceae*, *Ruminococcaceae*, and *Lachnospiraceae*, etc.), Bacteroidetes (*Prevotella*, *Bifidobacterium*, etc.), Proteobacteria (β*-Proteobacteria* and *Enterobacteriaceae*) and Actinobacteria (Yang et al., 2018; Jiang et al., 2019; Zhan et al., 2019). In the mammalian gastrointestinal tract, Bacteroidetes and Firmicutes phyla are the most abundant phyla. Several bacteria in these phyla have been confirmed to play vital roles in cognitive impairment (Jiang et al., 2019). An increase in the F/B ratio (relative abundance ratio of Firmicutes to Bacteroidetes) reflects an imbalance in gut microbiota composition (Magne et al., 2020). Although the phylum Proteobacteria is less abundant, it is gradually accepted that the prevalence of Proteobacteria and one of its families, *Enterobacteriaceae*, is also an important marker for gut dysbiosis (Shin et al., 2015; Rivera-Chavez et al., 2017).

In the present study, 16S rRNA, Firmicutes, Bacteroidetes, several classes of Proteobacteria and *Enterobacteriaceae*, were detected using qPCR. We found that anesthesia and surgery significantly decreased the content of β*-Proteobacteria* (a class of phylum Proteobacteria) and increased the content of Bacteroidetes, γ/δ*-Proteobacteria*, and ε*-Proteobacteria*. However, cefazolin could reverse these AS-induced changes. These results suggested that the disruptions of Bacteroidetes and Proteobacteria phyla might be an important microbial signature of POCD and modulating the two phyla is an approach for improving POCD.

Liang et al. (2018) found that cefazolin alone reduced the expression of 16S rRNA (1 day after cefazolin administration) but this change disappeared after 2 weeks of recovery. Moreover, surgery delayed the recovery of cefazolin-induced disturbance. In our study, 16S rRNA was not significantly changed in cefazolin groups with surgery at 10 days after cefazolin administration. In the present study, besides 16S rRNA, specific bacteria were further detected. There was no difference in Bacteroidetes and Firmicutes after cefazolin administration without surgery. Interestingly, cefazolin alone decreased the expression of β*-Proteobacteria* and increased expressions of γ/δ*-Proteobacteria* and *Enterobacteriaceae*. Given that the cefazolin alone group showed worse performance, the disturbance of Proteobacteria phyla might play a more important role in cognitive dysfunction.

Gut microbiota-derived metabolites, including nutrients, vitamins, neurotransmitters, and hormones, participate in various physiological processes (Schroeder and Backhed, 2016; Han et al., 2021). However, the roles of microbiota-derived metabolites in POCD have been rarely studied. SCFAs, the important microbiota-derived metabolites produced by fermentation of dietary fiber, have been shown to influence cognitive processes via multiple mechanisms, including affecting immune responses and neuroinflammation, modulating secretion of gut-derived hormones and neurotransmitters and activating vagal afferents (Dalile et al., 2019). Acetic, propionic and butyric acids are the most abundant SCFAs while other SCFAs, such as valeric and hexanoic acids, are less abundant. A previous study found that the occurrence of gut dysbiosis altered the levels of SCFAs (Lee et al., 2020). The contents of plasma SCFAs were measured in the present study. Our results showed that the contents of acetic, propionic, isobutyric and butyric acids accounted for 76.5–93.1% of total SCFAs (Figure 6I). AS and cefazolin alone reduced total SCFAs and almost all types of SCFAs. This indicated that a decrease in SCFAs might contribute to POCD. However, perioperative administration of cefazolin reversed the decrease in total SCFAs mainly through increasing the contents of plasma acetic and propionic acids. These findings suggested that modulating SCFAs (mainly acetic and propionic acids) might be involved in the enhancing effects of cefazolin on anesthesia and surgery-induced cognitive impairments.

Emerging evidence has shown that SCFAs, especially propionic acid, may modulate BBB function (Hoyles et al., 2018; van der Hee and Wells, 2021). An *in vitro* study demonstrated that propionate bound to its receptor free fatty acid receptors (FFAR) 3 on brain endothelium, activated the nuclear factor erythroid 2-related factor 2 (Nrf2) signaling pathway and protected BBB from oxidative stress (van der Hee and Wells, 2021). Given that BBB disruption was improved by cefazolin in our study, these results suggested that perioperative use of cefazolin might ameliorate POCD via increasing plasma levels of propionic acid and activating FFAR3/Nrf2 signaling in brain endothelium.

This study has several limitations. First, we did not examine the specific SCFAs-producing bacteria, which would help understand the relationships between gut microbiota and SCFAs after cefazolin administration. It is necessary to explore how cefazolin changes the specific microbiome in the future. Second, although various previous studies support our hypothesis and conclusions, we did not prove the causality of alterations in microbiota, SCFAs, BBB function and cognitive function, which warrants further studies. Third, we did not measure the levels of fecal SCFAs. A large amount of SCFAs is utilized by colonic epithelial cells. Therefore, analyses of fecal samples should be conducted to further understand the interplay between gut microbiota and associated metabolites. Lastly, because we separated the brain of each mouse into two halves for Western blot and immunochemistry assays, possible differences between bilateral hippocampus were not considered.

In summary, we provide novel evidence that perioperative use of cefazolin is beneficial to POCD. Moreover, cefazolin improved anesthesia and surgery-induced cognitive impairments by modulating BBB permeability, gut bacteria composition and plasma levels of SCFAs. However, cefazolin alone induced gut dysbiosis and cognitive impairment in healthy aged mice. Therefore, cefazolin should be considered for clinical prevention POCD but its use should be carefully controlled in the elderly.

## Data Availability Statement

The raw data supporting the conclusions of this article will be made available by the authors, without undue reservation.

## Ethics Statement

The animal study was reviewed and approved by the Experimental Animal Care and Use Committee of Tongji Hospital, Tongji Medical College, Huazhong University of Science and Technology.

## Author Contributions

AL obtained funding. AL, DH, and ShaL designed the study and wrote the manuscript. DH and ShaL performed the behavioral assessment, molecular biological detections, and data analyses. XW and ZX helped to conduct the behavioral tests. XW, ZX, and ShiL participated in statistical analyses and helped draft the manuscript. All authors read and approved the final manuscript.

## Conflict of Interest

The authors declare that the research was conducted in the absence of any commercial or financial relationships that could be construed as a potential conflict of interest.

## Publisher’s Note

All claims expressed in this article are solely those of the authors and do not necessarily represent those of their affiliated organizations, or those of the publisher, the editors and the reviewers. Any product that may be evaluated in this article, or claim that may be made by its manufacturer, is not guaranteed or endorsed by the publisher.

## Abbreviations

AS: anesthesia and surgery
EB: Evans blue
HC: healthy control
MWMT: Morris water maze test
NCD: non-cognitive dysfunction
OCLN: occludin
OFT: open field test
POCD: postoperative cognitive dysfunction
SCFAs: short chain fatty acids
ZO-1: zonula occludens-1.
