# Supplementary material for: Cefazolin Improves Anesthesia and Surgery-Induced Cognitive Impairments by Modulating Blood-Brain Barrier Function, Gut Bacteria and Short Chain Fatty Acids
Source: Front Aging Neurosci. 2021 Oct 13;13:748637. doi: 10.3389/fnagi.2021.748637 (PMC8548472; doi:10.3389/fnagi.2021.748637)
Supplement: Supplementary file 1 [file Presentation_1.pptx]

## Slide 1
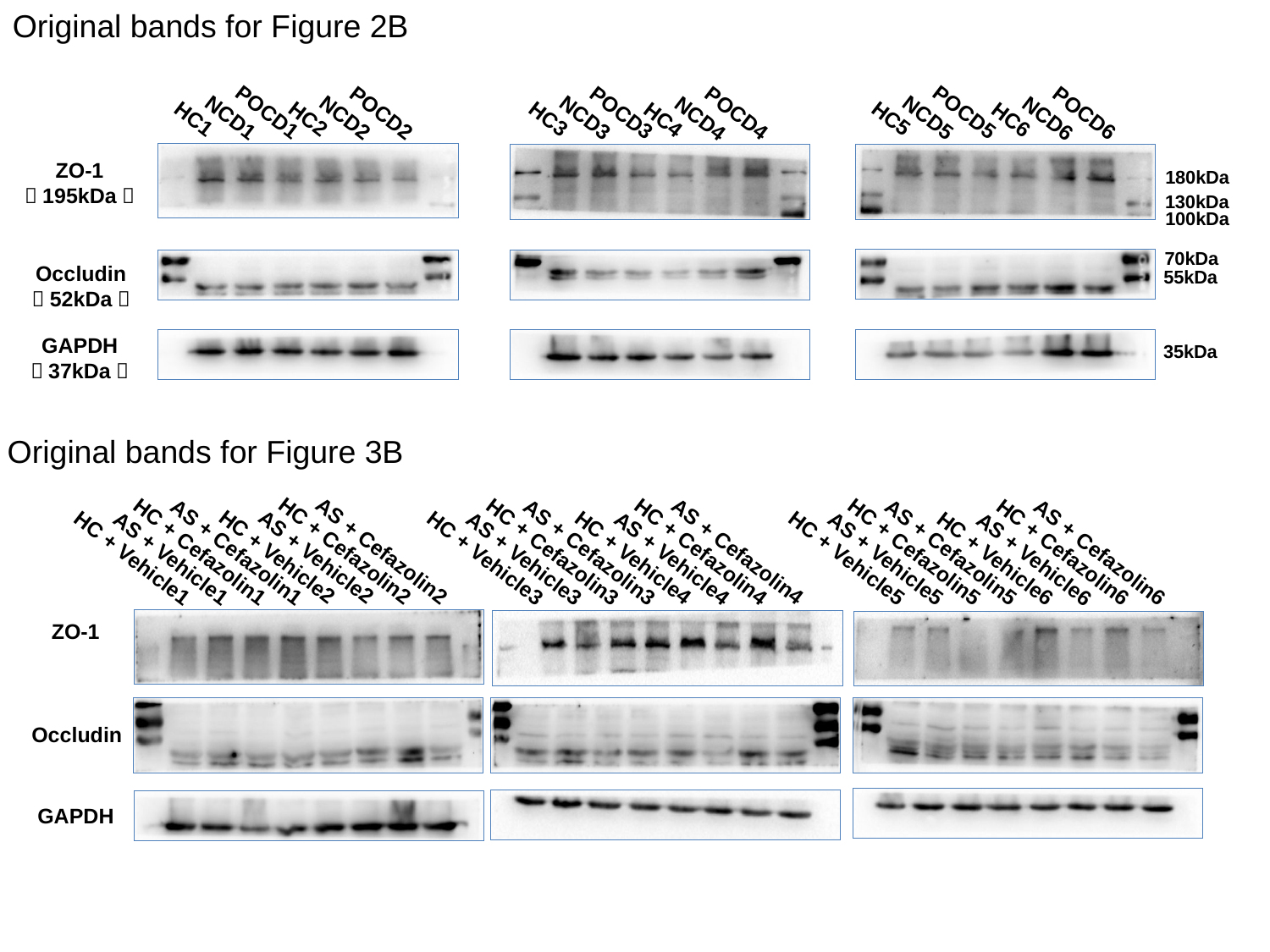

Original bands for Figure 2B
POCD5
NCD5
HC5
POCD1
NCD1
HC1
POCD3
NCD3
HC3
POCD2
NCD2
HC2
POCD6
NCD6
HC6
POCD4
NCD4
HC4
ZO-1
（195kDa）
180kDa
130kDa
100kDa
70kDa
Occludin
（52kDa）
55kDa
GAPDH
（37kDa）
35kDa
Original bands for Figure 3B
HC + Cefazolin2
HC + Vehicle2
AS + Vehicle2
AS + Cefazolin2
HC + Cefazolin4
HC + Vehicle4
AS + Vehicle4
AS + Cefazolin4
HC + Cefazolin3
HC + Vehicle3
AS + Vehicle3
AS + Cefazolin3
HC + Cefazolin1
HC + Vehicle1
AS + Vehicle1
AS + Cefazolin1
HC + Cefazolin5
HC + Vehicle5
AS + Vehicle5
AS + Cefazolin5
HC + Cefazolin6
HC + Vehicle6
AS + Vehicle6
AS + Cefazolin6
ZO-1
Occludin
GAPDH
